# In silico designing and optimization of anti‐epidermal growth factor receptor scaffolds by complementary‐determining regions‐grafting technique

**DOI:** 10.1002/qub2.63

**Published:** 2024-07-10

**Authors:** Razieh Rezaei Adriani, Seyed Latif Mousavi Gargari, Hamid Bakherad, Jafar Amani

**Affiliations:** ^1^ Department of Biology Shahed University Tehran Iran; ^2^ Department of Pharmaceutical Biotechnology School of Pharmacy and Pharmaceutical Sciences Isfahan University of Medical Sciences Isfahan Iran; ^3^ Applied Microbiology Research Center System Biology, and Poisonings Institute Baqiyatallah University of Medical Sciences Tehran Iran

**Keywords:** CDR grafting technique, EGFR, molecular dynamic simulation, panitumumab

## Abstract

Monoclonal antibodies are attractive therapeutic agents in a wide range of human disorders that bind specifically to their target through their complementary‐determining regions (CDRs). Small proteins with structurally preserved CDRs are promising antibodies mimetics. In this in silico study, we presented new antibody mimetics against the cancer marker epidermal growth factor receptor (EGFR) created by the CDRs grafting technique. Ten potential graft acceptor sites that efficiently immobilize the grafted CDR loops were selected from three small protein scaffolds using a computer. The three most involved CDR loops in antibody‐receptor interactions extracted from panitumumab antibody against the EGFR domain III crystal structure were then grafted to the selected scaffolds through the loop randomization technique. The combination of three CDR loops and 10 grafting sites revealed that three of the 36 combinations showed specific binding to EGFR DIII by binding energy calculations. Thus, the present strategy and selected small protein scaffolds are promising tools in the design of new binders against EGFR with high binding energy.

## 1 INTRODUCTION

Antibodies, ∼150 kDa glycoproteins, consist of two light and two heavy polypeptide chains with six complementarity‐determining regions (CDRs) for specific binding to their targets [1]. However, their therapeutic use is limited due to the low tissue penetration. Recently, advances in the structural engineering of proteins led to the construction of many small‐size proteins (3–20 kDa) with the ability to bind to epitopes recognized by mono antibodies [2]. Among all the engineered and recombinant proteins, single chain variable fragments (scFvs) composed of VH and VL, with the same antigen‐binding specificity can retain the affinity of a whole antibody molecule [3]. Low molecular weight, high rate of excretion, good tissue penetration, and low costs for production of these antibody mimetics converted them into a promising alternative for targeted therapies [4]. In recent years, small non‐immunoglobulin proteins have been produced, using techniques such as affinity selection from protein libraries and antibody fragmentation technologies. Nonetheless, these methods are currently constrained in their ability to produce fragments of up to 30 kDa [4]. One effective approach for creating receptor‐binding proteins with enhanced affinity involves designing virtual small protein fragments obtained from extensive libraries [4]. Conversely, the length, conformation, and the conserved amino acid residues of the hypervariable CDRs loop are pivotal in defining the native binding site [5]. By employing the CDRs grafting strategy, protein scaffolds featuring variable loops have been chosen to offer a structural framework that support the grafted CDRs. In line with this approach, Nicaise et al. [6] devised protein scaffolds against lysozyme, integrating the same CDRs from a specific antibody. However, the designed scaffolds exhibited varying affinities toward lysozyme, highlighting that the selection of scaffolds directly affects the affinity of grafted CDRs. Therefore, for designing antibody mimetics, selecting proper protein scaffolds and determining appropriate CDRs peptide sequences is essential [7]. Among many introduced non‐immunoglobulin scaffolds, anticalins with a smaller size of 180 amino acids consisting of one polypeptide chain, a beta‐sheet structure, and four flexible loops without any glycosylated structure can be produced in prokaryotic hosts [8]. Another example of protein scaffolds is the 10th domain of human fibronectin type III (Fn3, also called Adnectin). Adnectin is a 94‐residue monomeric protein with a β‐sandwich fold structure and six internal loops without disulfide bonds. This protein mimics the interaction of CDRs with the target through three loops on the tip of molecules [8]. The third structure selected for this study is VHH, with small molecular weight, low aggregation, and high solubility, which contains three variable loops [5]. This existing assortment represents an ideal CDR acceptor repertoire for our approach. In addition to the structure of the CDRs acceptor, the framework plays a vital role in CDR orientation and conformation. Therefore, framework residues should be considered in designing a protein scaffold with high binding affinity to the target [9]. Here, we aimed to simulate the CDRs conformation with two residues per side to keep the framework of the native structure in three selected scaffolds of humanized VHH, Fn3, and lipocalin with at least three flexible loops for grafting CDRs isolated from the target antibody. Three CDRs peptides of H2, H3, and L3 that play an important role in the recognition of the target were extracted from the monoclonal antibody panitumumab that binds specifically to the epidermal growth factor receptor (EGFR) DIII. These CDRs were then grafted through loop randomization in all these scaffolds. Apart from scFv, which carries all six CDRs in their native structure, 36 anti‐EGFR DIII protein candidates were created (24 candidates from lipocalin scaffold, six structures of Fn3, and six candidates of humanized VHH). Among all these structures, three of them were selected for further analysis in molecular dynamic simulation.

## 2 RESULTS

### 2.1 Strategy for creating anti‐EGFR scaffolds

**FIGURE 1:**
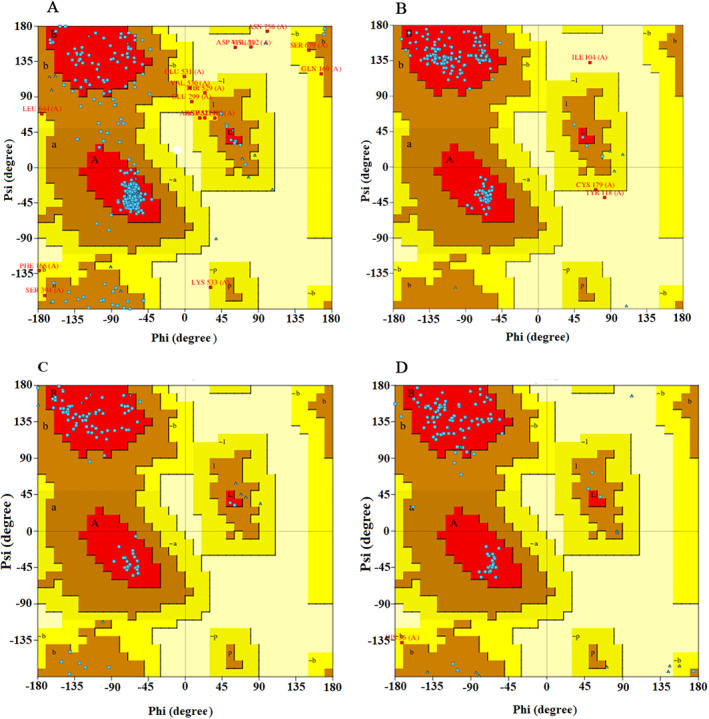
Ramachandran plots of generated models by Modeller software. (A) ScFv structure. (B) Scaf1. (C) Scaf2. (D) Scaf3. ScFv, single chain variable fragments.

Selecting an appropriate scaffold for grafting effective CDRs in binding is crucial to mimic the antibody‐receptor interaction. Accordingly, we designed small virtual antibody mimetics with high affinity against EGFR DIII based on the panitumumab antibody. Ideal traits of scaffold candidates as CDRs acceptors include a suitable size (<180 amino acids) with a minimum of three variable loops, low disulfide bonds, and non‐immunogenicity. Then, active CDRs in ligand‐receptor interaction were determined and immobilized randomly into variable loops of each scaffold. All designed structures were analyzed (Table 1) and after docking against EGFR DIII, the best ones from each scaffold were selected for Molecular Dynamic (MD) simulation analysis. The Ramachandran plots of scFv and three selected scaffolds are depicted in Figure 1, and the selected scaffolds’ residue information is shown in Table 2.

**TABLE 1:** Quality assessment of generated models by Modeller.

Scaffold	Number of variable loops	Number	Designed structures	DOPE score	*G* factor	*Z* score PROSA
ScFv	‐	1	‐	−53314.67188	−0.04	−5.34
3BX7	4	1	L1:H2‐H3‐L3	−19555.44336	−0.18	−5.95
		2	L1:H2‐L3‐H3	−19259.66602	−0.15	−5.9
		3	L1:H3‐H2‐L3	−19416.04492	−0.21	−5.6
		4	L1:H3‐L3‐H2	−19294.21094	−0.17	−5.83
		5	L1:L3‐H2‐H3	−19429.92969	−0.29	−5.67
		6	L1:L3‐H3‐H2	−19325.28906	−0.18	−5.64
		7	L2:H2‐H3‐L3	−20591.39453	−0.22	−5.79
		8	L2:H2‐L3‐H3	−20542.80469	−0.11	−6.3
		9	L2:H3‐H2‐L3	−20866.83789	−0.08	−6.01
		10	L2:H3‐L3‐H2	−20605.34961	−0.09	−6.13
		11	L2:L3‐H2‐H3	−20938.12500	−0.15	−6.18
		12	L2:L3‐H3‐H2	−20553.53711	−0.08	−6.05
		13	L3:H2‐H3‐L3	−20396.19727	−0.07	−6.44
		14	L3:H2‐L3‐H3	−20474.53320	−0.16	−6.15
		15	L3:H3‐H2‐L3	−20461.74023	−0.09	−6.23
		16	L3:H3‐L3‐H2	−20756.90625	−0.10	−6.2
		17	L3:L3‐H2‐H3	−20462.06250	−0.13	−6.31
		18	L3:L3‐H3‐H2	−20660.40625	−0.06	−6.22
		19	L4:H2‐H3‐L3	−20999.69141	−0.05	−5.78
		20	L4:H2‐L3‐H3	−21099.89648	−0.18	−5.63
		21	L4:H3‐H2‐L3	−20620.75781	−0.07	−5.78
		22	L4:H3‐L3‐H2	−21000.22852	−0.07	−6
		23	L4:L3‐H2‐H3	−20613.94141	−0.19	−5.23
		24	L4:L3‐H3‐H2	−20824.11133	−0.11	−5.66
1TTF	3	25	H2‐H3‐L3	−9152.06445	−0.25	−3.91
		26	H2‐L3‐H3	−9291.40234	−0.24	−3.6
		27	H3‐H2‐L3	−9089.15918	−0.19	−3.86
		28	H3‐L3‐H2	−9045.37598	−0.19	−3.87
		29	L3‐H2‐H3	−9196.52051	−0.43	−3.35
		30	L3‐H3‐H2	−9074.09375	−0.35	−3.7
3dwt	3	31	H2‐H3‐L3	−10967.31934	−0.26	−4.63
		32	H2‐L3‐H3	−10813.43457	−0.24	−4.76
		33	H3‐H2‐L3	−9031.69922	−0.4	−4.17
		34	H3‐L3‐H2	−8909.66797	−0.35	−4.04
		35	L3‐H2‐H3	−6832.06787	−0.13	−1.89
		36	L3‐H3‐H2	−7095.58789	−0.13	−1.26

*Note*: Three highlighted scaffolds 18, 28, and 36 refer to Scaf1, Scaf2, and Scaf3, respectively.

Abbreviation: ScFv, single chain variable fragments.

**TABLE 2:** Residue information of four selected structures.

	ScFv		Scaf1		Scaf2		Scaf3	
Residue information	No. of residue	Value %	No. of residue	Value %	No. of residue	Value %	No. of residue	Value %
Most favored regions	615	90.3	150	93.8	77	89.5	88	86.3
Additional allowed regions	50	7.3	7	4.4	9	10.5	13	12.7
Generously allowed regions	13	1.9	1	0.6	0	0.0	1	1.0
Disallowed regions	3	0.4	2	1.2	0	0.0	0	0.0
Non‐glycine and non‐proline residues	681	‐	160	‐	86	‐	102	‐
End‐residues (excl. gly and pro)	2	‐	1	‐	2	‐	2	‐
Glycine residues	58	‐	11	‐	9	‐	13	‐
Proline residues	27	‐	10	‐	5	‐	3	‐

Abbreviation: ScFv, single chain variable fragments.

### 2.2 Molecular docking analysis

To screen newly designed scaffolds targeting the EGFR on the surface of cancer cells, a molecular docking study was performed using the HADDOCK webserver (Table 3). The results showed the binding of the ligands in the same position and orientation with acceptable binding scores as compared to the reference complexes. It verified that the selected docking parameters were optimal. Docked scaffolds are ranked based on their docking scores. Docking results revealed that three selected scaffolds out of 36 had docking scores between the range of −99 to −145.8 in which Scaf1 showed the best docking score in comparison to scFv and 5SX4 complex. The complex of all three selected scaffolds and the reference of scFv with EGFR DIII were analyzed in MD analysis.

**TABLE 3:** Various energies of references and designed structures calculated by HADDOCK.

Structure	HADDOCK score	Cluster size	RMSD	Van der Waals energy (kJ/mol)	Electrostatic energy (kJ/mol)	Desolvation energy (kJ/mol)	Restraints violation energy (kJ/mol)	BSA	*Z*‐Score
Reference (5SX4)	−122.83 ± 3.6	384	1.05 ± 0.6	−63.7 ± 0.7	−211.35 ± 37.6	−17.35 ± 7.4	4.2 ± 1.47	1881.0 ± 97.1	−1.4
scFv	−123.8 ± 2.9	17	15.5 ± 0.1	−83.0 ± 6.9	−295.3 ± 71.4	4.6 ± 9.1	137.5 ± 29.09	2708.2 ± 69.5	−1.3
Scaf1	−145.8 ± 6.0	371	16.4 ± 0.2	−76.8 ± 4.4	−230.5 ± 37.0	−36.0 ± 7.9	131.0 ± 9.84	2462.5 ± 62.5	−1.4
Scaf2	−103.6 ± 2.4	152	1.0 ± 0.6	−59.4 ± 3.5	−169.8 ± 21.9	−30.6 ± 3.3	202.6 ± 11.40	1822.9 ± 65.8	−1.9
Scaf3	−99.0 ± 4.1	14	0.7 ± 0.4	−65.3 ± 8.8	−370.3 ± 41.1	0.0 ± 5.1	403.4 ± 16.19	2171.4 ± 139.1	−1.6

*Note*: RMSD from the overall lowest‐energy structure.

Abbreviations: BSA, buried surface area; RMSD, root mean square deviation; ScFv, single chain variable fragments.

### 2.3 Molecular dynamics simulation

**FIGURE 2:**
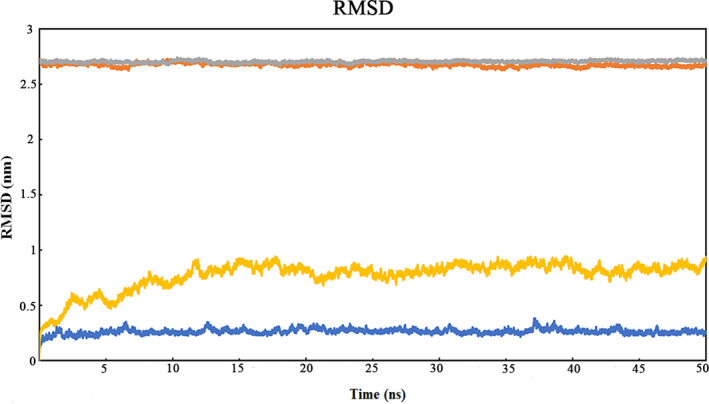
RMSD profile of all protein‐receptor complexes for 50 ns MD simulation period. scFv‐EGFR DIII (Orange), Scaf1‐EGFR DIII (Gray), Scaf2‐EGFR DIII (Blue), and Scaf3‐EGFR DIII (Red). EGFR, epidermal growth factor receptor; MD, molecular dynamic; RMSD, root mean square deviation; ScFv, single chain variable fragments.

**FIGURE 3:**
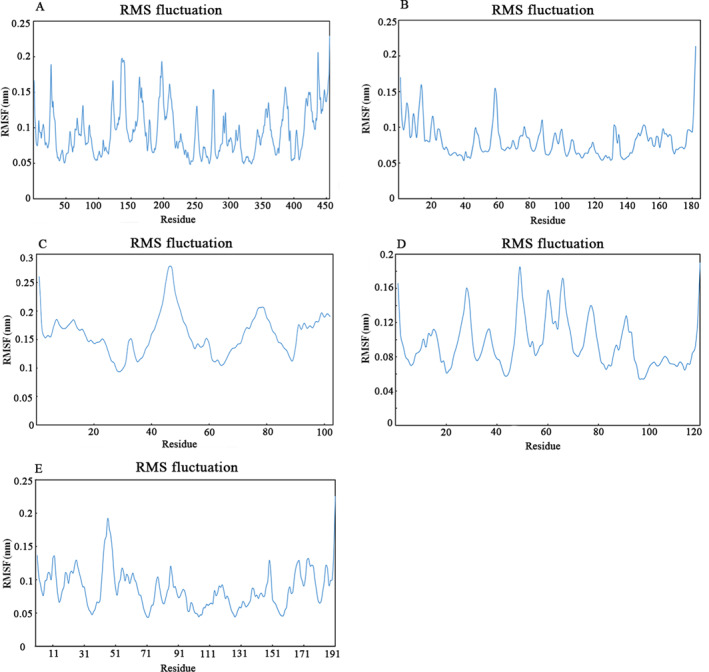
The graph representing the RMSF values of Cα atoms for 50 ns trajectories. (A) ScFv structure. (B) Scaf1. (C) Scaf2. (D) Scaf3. (E) EGFR DIII. EGFR, epidermal growth factor receptor; RMSF, root mean square fluctuation; ScFv, single chain variable fragments.

The molecular dynamic method was used to analyze the physical movements of atoms and molecules and to study conformational change at the atomic level. To assess the binding stability and determine binding‐free energy against the EGFR active site, three scaffolds and scFv were subjected to 50 ns molecular dynamic simulation. Further, all four scaffolds were analyzed by root mean square deviation (RMSD), root mean square fluctuation (RMSF), H bond, and molecular mechanics Poisson–Boltzmann surface area (MM‐PBSA) calculations to examine the protein stability and dynamic behavior throughout the simulation period. The variation of all the scaffolds‐receptor complex was determined by the RMSD during the 50 ns MD simulation. The RMSD of alpha carbon was calculated (Figure 2), and results indicated that all four complexes remained stable throughout the simulation with an average RMSD of 0.75, 2.65, 0.2, and 2.7 for scFv (Orange), Scaf1 (Gray), Scaf2 (Blue), and Scaf3 (Red), respectively. A slight fluctuation in the case of the scFv complex was noted during the first 6 ns, which achieved the equilibrium and remained stable throughout the simulation. Measuring the average movement of the atom position at the specific temperature and pressure was performed by RMSF analysis. The fluctuations in the constituents’ residues were observed for all four structures and plotted to compare the flexibility of each residue in complexes (Figure 3). RMSF was calculated for EGFR DIII, scFv, and three selected scaffolds. Low RMSF values obtained for all four complexes indicate the good stability of the system. Although there are little fluctuations in some residues of the scFv and Scaf2, all grafted residues in designed scaffolds had low RMSF values. Otherwise, the fluctuation during all four CDR loops‐receptor interactions was below 0.2 nm, which is perfectly acceptable and shows the good stability of designed structures.

### 2.4 Hydrogen bonding analysis

**FIGURE 4:**
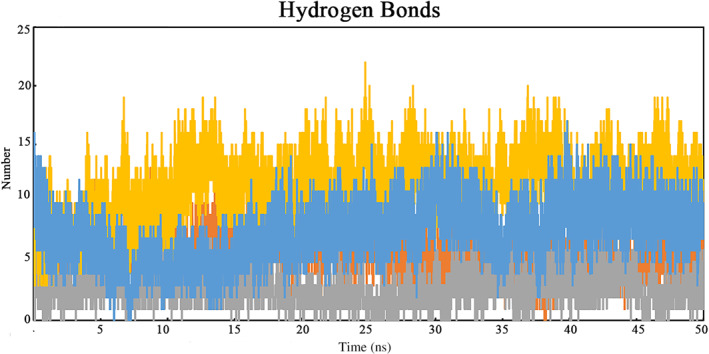
Diagram representing the dynamics observed in the hydrogen bonding patterns for scFv‐EGFR as the reference molecule (Orange), Scaf1‐EGFR DIII (Gray), Scaf2‐EGFR DIII (Blue), and Scaf3‐EGFR DIII (Red). EGFR, epidermal growth factor receptor; ScFv, single chain variable fragments.

Hydrogen bonding between a ligand and receptor stabilizes their interaction [10]. It also shows the specificity, metabolism, and adsorption of the drug. To further explain the conformational stability, the total number of hydrogen bonds between each scaffold‐receptor was analyzed (Figure 4). This indicated that the scFv and EGFR DIII complex shows strong bonding interaction throughout the 50 ns of MD simulations. Analyzing the reference complex of scFv with the receptor, around 13 hydrogen bonds (Orange) were observed in the complex. At the same time, 13, 5, and 4 residues in Scaf1 (Gray), Scaf2 (Blue), and Scaf3 (Red) were involved in hydrogen interaction with the receptor. The above‐detailed H‐bond analysis showed that Scaf1 was bound to the EGFR DIII as effectively and tightly as the reference molecule scFv.

### 2.5 Molecular interaction of the top scaffolds with EGFR

**FIGURE 5:**
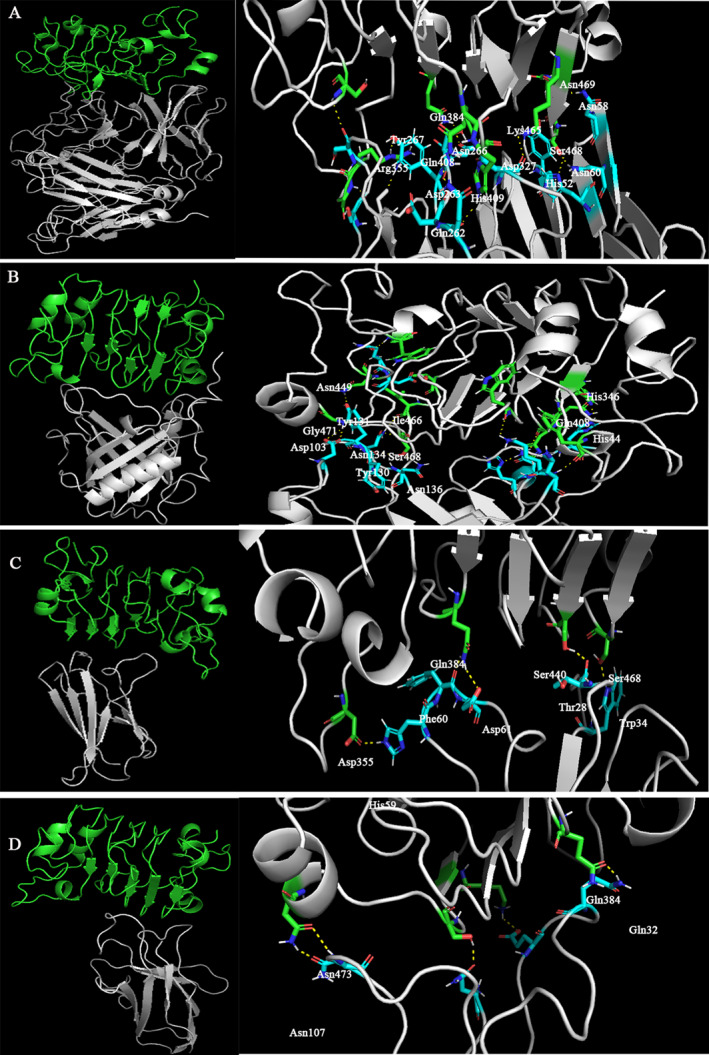
3D structures of scFv and three top ligands in complex with EGFR. (A) ScFv‐EGFR DIII complex and hydrogen bonds in CDR regions. (B) Scaf1‐EGFR DIII complex and hydrogen interaction in three inserted CDR loops. (C) Scaf2‐EGFR DIII complex and its hydrogen interactions in grafted CDRs. (D) Scaf3‐EGFR DIII complex and residues involved in hydrogen bonding. CDR, complementary‐determining region; EGFR, epidermal growth factor receptor; ScFv, single chain variable fragments.

The 3D interaction of the top 3 scaffolds and the reference molecules of the panitumumab and scFv were visualized by PyMOL software [11]. The docked poses demonstrated that all six CDR loops in the panitumumab structure interacted through 12 hydrogen bonds with EGFR D3. Asp50, Phe91, Asp92, Lue94 in light chain and Asp33, Tyr35, Tyr55, Asn58, Thr59, Asp100, and Thr103 in heavy chain are involved in H bonds. Analyzing the scFv complex showed that CDR regions H1, H2, L1, L2, and L3 interacted with EGFR by several residues, among which Tyr35, Asn58, and Asp327 are common to the panitumumab complex. Selected scaffolds interacted with the receptor through hydrogen bonds of residues in L3, H3, and H2 CDR loops in Scaf1, H3, L3 in Scaf2, and L3, H2 in Scaf3 (Figure 5). Analyzing these three scaffolds indicated that Tyr131, Asn134 in Scaf1, Thr28, Phe60, Asp61 in Scaf2, and Asn107 are in common with reference molecules. These scaffolds also form hydrophobic contacts with EGFR DIII, which are presented in Table 4. From the visualization study of all three scaffolds, we observed that the L3 CDR loop involved H‐bonding interactions similar to the reference molecules. It suggests that this loop plays an important role in scaffold‐receptor hydrogen interaction. From the findings, it can be seen that Scaf1 mimics the conformation of the protein‐receptor complex with all three CDR loops involved.

**TABLE 4:** Interaction of reference 5SX4, scFv, and top three hits with EGFR domain III.

Scaffold	Active residues of the receptor (EGFR)	Residues of ligand	Common active site hydrogen bond residues in CDR loops	Hydrophobic contacts in CDR regions
Panitumumab	K465/I466 Lys465 Ser468 Asn469 .......... Gln408 Ser440/Ser418 Asn384 Lys443/Asp420 Lys443 Ser468 Lys465	Light chain H: Tyr32 H: Asp50 H: Phe91, Asp92 H: Leu94 Heavy chain H: Asp33 H: Tyr35 H: Tyr54, Tyr55 H: Asn58 H: Thr59 H: Asp100 H: Thr103	Light chain Asp50, Tyr32, Phe91, Asp92, Leu94 Heavy chain Tyr35, Asp33, Thr103, Tyr55, Thr59, Asn58 Asp100	Light chain Asp50, Tyr32, Phe91, Asp92, Leu94 Heavy chain Tyr35, Asp33, Thr103, Tyr54, Val102, Tyr55, Thr59, Ser56, Asn60, Asn58
ScFv	Ser468 Lys465 Gln408 His409 Asn469 Gln384 Arg353 Gly354	H: His52, Tyr35, Asn60 H: Asp327 H: Asp263 H: Gln262 H: Asn58 H: Asn266 H: Tyr267, Ser287 H: Ser300	Tyr35, His52, Asn58, Asn60, Gln262, Asp263, Asn266, Tyr267, Asp327	Asn58, Phe326, Ile264, Asp263, Val102, Tyr54, His328, Asp327, Ser56, Asn288, Ser265, Thr103, Tyr267, Ser287, Tyr35, Asp285
Scaf1	Ile466, Ser468 Asn449 Ser468 Gly471 Gln408, His346 Thr464 His409 Phe352 Trp453 Trp386	H: Asn134 H: Tyr131 H: Tyr130, Asn136 H: Asp103 H: His44 H: Asp2 H: His169 H: Lys73 H: Gln1 H: Lys72	His44, Asp103, Tyr130, Tyr131, Asn134, Asn136	Tyr131, Asp103, Phe102, His41, Asp43, Phe42, Gln40, Asn134, Asn136, His44, Leu45, Pro46
Scaf2	Asp355 Ser468 Gln384 Ser440	H: His59 H: Trp34 H: Asp61, Phe60 H: Thr28	Thr28, Trp34, His59, Phe60, Asp61	Phe60, His59, Thr28, Gly29, Arg26, His62, Val27, Tyr89, Leu65, Phe31, Ala30
Scaf3	Ser468 Lys465 Gln384 Asn473 (2 h bonds)	H: Gln1 H: Glu29 H: Gln32 H: Asn107	Asn107	Phe34, Cys31, Ser105, Ile102, Tyr103, Phe30, Gly106, His101

Abbreviations: CDR, complementary‐determining region; EGFR, epidermal growth factor receptor; ScFv, single chain variable fragments.

### 2.6 Binding free energy

The binding free energy calculated from MD trajectories showed that the total binding energies of all complexes were observed in an acceptable range. The results of MM‐PBSA are given in Table 5. In particular, complex Scaf1‐EGFR DIII presented the lowest binding free energy and higher binding affinity with the receptor, suggesting a more stable protein conformation. These free energy calculations validated the molecular docking results, showing that this scaffold was favorably binding to EGFR DIII and can be determined as a suitable CDR acceptor to mimic the panitumumab‐EGFR interaction.

**TABLE 5:** MM‐PBSA results for all designed complexes.

Scaffold	Van der Waals energy (kJ/mol)	Electrostatic energy (kJ/mol)	Polar salvation energy (kJ/mol)	SASA energy (kJ/mol)	Binding energy (kJ/mol)
ScFv	−41.614 ± 88.530	−29.391 ± 76.994	134.397 ± 157.739	−6.290 ± 10.100	−26.406 ± 0.00
Scaf1	−0.681 ± 0.00	−12.649 ± 0.00	55.399 ± 0.00	−2.170 ± 0.00	−39.899 ± 0.00
Scaf2	−0.743 ± 0.00	−33.127 ± 1.876	47.807 ± 23.564	−2.567 ± 0.00	−9.476 ± 24.631
Scaf3	−0.393 ± 0.053	7.000 ± 8.667	−18.905 ± 70.590	−1.221 ± 3.377	−13.518 ± 72.532

Abbreviation: MM‐PBSA, molecular mechanics Poisson–Boltzmann surface area; ScFv, single chain variable fragments.

## 3 DISCUSSION

Antibody mimetics is a new technique based on highly structured scaffolds originating from natural proteins. Mutation in their key residues, especially in variable loops, can alter their affinity toward new targets [12]. In our research, we employed a CDR grafting strategy to design a total of 36 binders targeting the EGFR. This involved integrating CDR loops H2 and H3 from the heavy chain, as well as L3 from the light chain of panitumumab, which were found to have the most effective interactions with the receptor. These selected CDR loops were then grafted into new scaffolds. The extracellular domain 3 of EGFR was retrieved from EGFR/panitumumab complex (5SX4). It was then used as the target in our structure‐based virtual screening method for 36 CDR‐acceptors, designed by the loop alteration technique. In addition to the 36 designed binders, the scFv structure originating from panitumumab was docked against EGFR DIII. The primary criteria for selecting the binders were based on the docking scores and an analysis of the positions of CDR loops against the receptor. The docking scores and analysis of the CDR loop positions against the receptor were used as the main criteria for the binder selection. Further, the complexes of three protein‐EGFR DIII and scFv‐EGFR DIII were simulated through molecular dynamic analysis and their binding sites compared. The result was analyzed by different parameters such as RMSF, H‐bond, and MM‐PBSA. Therefore, the current study analyzed the behavior of the designed complexes using the protein‐receptor complex against domain III EGFR. It focused on the three top hits with the best docking scores that share a similar binding site to the reference molecules. The epidermal growth factor protein binding by EGFR occurs within a cleft between two extracellular domains, DI and DIII [13]. The binding site of panitumumab partially overlaps the Epidermal Growth Factor (EGF) binding site and prevents the dimerization of the EGFR ectodomain and its activation [14]. The overall binding of panitumumab to domain III is similar to cetuximab. Based on a mutational analysis, Voigt et al. [15] described critical amino acids overlap in panitumumab and cetuximab epitopes with the EGF binding site: K443 and D355 in panitumumab and D355, K443, Q408, H409, and S468 in the cetuximab binding site [16]. The panitumumab complex with EGFR DIII shows that upon binding, all six CDRs in the heavy and light chains interact without any conformational alteration. According to previous studies, CDRs L3, H2, and H3 commonly make the largest contribution to antigen binding [9]. The L3 CDR lines up adjacent to the final B‐strand of domain III with two interactions of D92 and L94. The heavy chain showed most of the specific hydrogen bond interactions made by H2 and H3. Most notably, three tyrosine residues interacted with the B‐strand of domain III at the same position as EGF. Y54, Y55, N58, and T59 in H2 interacted with N384, D420, and K443, respectively, while in H3, D100 has a water‐mediated hydrogen bond with S468. H3 makes an additional hydrogen bond between T103 and K465 of domain III [14]. It can be visualized that interaction with S468 had the highest interaction rate (Figure 5). This residue interacts with EGF, panitumumab, cetuximab, scFv, and all three designed scaffolds. The scFv structure interacted with critical residues of S468, Q408, and H409 through five H‐bonds in CDR regions, which overlap with the cetuximab binding site. Scaf1 interacted with the same epitope as cetuximab through three H‐bonds in inserted CDR loops against S468 and Q408. Scaf2, in addition to S468, interacted with D355, which is involved in both panitumumab and cetuximab binding sites. It indicates that this scaffold mimics the function of these two antibodies. In contrast to the other two scaffolds, Scaf3 could not make any hydrogen interaction in CDR regions with critical residues in the panitumumab binding site. However, Q1 in Scaf3 interacted with S468 and made hydrophobic contact with F30 against F412 in the panitumumab binding site. It shows that Scaf3 can be used as an alternative to panitumumab but with lower binding affinity. Therefore, all three scaffolds that exhibit the interaction with the same amino acid residues (S468, D355, Q408, and H409) are subjected to a 50 ns simulation process. The MD results confirmed the stability of scFv and three selected scaffolds throughout the simulation. The RMSF was calculated for domain III of EGFR, scFv, and three designed binders. It refers to the stability of the complex as high fluctuations related to more flexible and unstable bounds. Although there are some fluctuations in Scaf2 amino acid residues, they are not at the inserted CDR loops nor involved in protein interaction. The fluctuation during all interactions was below 0.2 nm, which is totally acceptable. To validate the docking energy of the protein‐receptor complex, an MM‐PBSA calculation was performed. The designed structures presented comparatively acceptable MM‐PBSA scores compared to the scFv structure. The calculated binding free energy of these binders was −26.4, −39.89, −9.47, and −13.51 kJ/mol for scFv, Scaf1, Scaf2, and Scaf3, respectively. Therefore, they represent excellent candidates for further investigation in vitro analysis. The only exception is the Scaf3 complex, which showed a slightly lower MM‐PBSA value in accordance with its visual analysis results. By analyzing the binding energy and stability through dynamic simulation, we have shown that scFv, Scaf1, and Scaf2 may be potential binders that mimic the function of panitumumab. We tested in vitro and in vivo function of the scFv structure against EGFR, which confirmed the results of the in silico report. From this study, Scaf1 and Scaf2 showed promising high affinity against EGFR in comparison to 5SX4 and scFv complexes. Thus, the results of this study indicate that the anti‐EGFR activity of these compounds could be significantly effective against highly overexpressed EGFR cancers. These detailed analyses indicate that computational antibody mimetics through the CDR grafting technique is very efficient. This in silico study may offer the opportunity to explore these scaffolds in vitro against EGFR.

## 4 MATERIALS AND METHODS

### 4.1 Identification of antigen‐binding CDRs

The structure of the EGFR DIII/Panitumumab complex (PDB entry 5SX4) was used as the initial complex for extracting the effective CDRs in interaction with the receptor. Two CDRs, H2, and H3, from the heavy chain and L3 from the light chain, were selected for grafting into three scaffolds of lipocalin (PDB entry 3BX7), Fn3 (1TTF), and VHH (3dwt). The sequence of these CDRs is defined by Parapred software [17], each with two residues per site (residues on the upper and lower core of variable domains affecting CDRs conformation) [9]. To accomplish this, we obtained the sequences of both the heavy and light chains of panitumumab, which include the variable domains, from PDB ID 5SX4. We then defined the CDRs residues based on the Chothia definition, using the ANARCI software (Figure S2). ANARCI is a valuable tool that classifies and assigns numbers to antibody variable domain sequences. It can annotate residue positions based on the location of regions of high sequence variation between sequences of the same domain type.

### 4.2 Identification of grafting loops

The initial coordinates of three selected graft acceptors were taken from PDB accession codes 3BX7 (Scaf1), 1TTF (Scaf2), and 3dwt (Scaf3) (Figure S1). Three‐dimensional models of 36 designed structures after immobilizing CDR peptides into variable loops were generated by Modeller software (Mod10.2). The best models with the lowest DOPE score from each 36 designed scaffolds were selected for further analysis. The scFv model of the panitumumab antibody was extracted from 5SX4 PDB entry and generated as the second reference molecule alongside the whole structure of the antibody. It was used for comparing binding energies. The quality of models was assessed by online web servers, including PDBSum and ProSA‐web [18]. The model structures were evaluated by Ramachandran plot assessment using the PDBSum server.

### 4.3 Docking

To find potential candidates against the EGFR DIII, molecular docking was carried out to screen conformations of designed ligands with high stability at the binding site of EGFR DIII by the HADDOCK 2.2 web server [19]. Initially, molecular docking analysis was performed with the reference molecules in the active site of EGFR to reproduce the same conformation, similar to the co‐crystallized ligand (5SX4). The result of scFv docking with EGFR DIII was also used as the second reference molecule. To set the docking constraints, the CDRs interacting residues of EGFR DIII were defined as active residues [20], while all other solvent‐accessible residues were defined as passive residues. To drive the docking simulation, the information on user‐defined docking constraints was converted into ambiguous interaction restraints by HADDOCK. The docking protocol consists of three steps: a rigid body energy minimization, a semi‐flexible refinement in the torsion angle, and finally refinement in the explicit solvent. A maximum of 200 water‐refined models obtained in the first stage of the docking run were clustered using a pair‐wise main chain RMSD cut‐off of 7.5 and a minimum cluster size of 4 as criteria. Several clusters were generated. These clusters were ranked based on the HADDOCK scores calculated on the basis of weighted inter‐molecular energy terms. The best‐refined model from the highest‐ranked cluster obtained from the docking run was used as the HADDOCK representative structure. Among all generated receptor‐ligand complexes, models with lower binding energy were selected for MD simulation.

### 4.4 Molecular dynamic simulation

The MD simulation is widely used to determine the structural stability of the protein and protein‐ligand complexes under physiological conditions [14]. To evaluate the stability of two obtained structures from docking analysis, molecular dynamic simulation was applied using GROMACS 4.6.5 suits [21]. GROMOS96 force field and simple point charge as the water model were used to generate topology files. All simulations were analyzed at 343 K. To neutralize each system, some water molecules were randomly replaced by ions. After being neutralized, the system’s energy was minimized using the steepest descent algorithm. Then, all systems were simulated for 100 ps under NVT at 300 K and NPT ensembled at the pressure of 1 bar within periodic boundary conditions and restraint force of 100 kJ/mol using modified Berendsen thermostat and Parinello‐Rahman barostat algorithms, respectively. The electrostatic interactions were evaluated using the Particle Mesh Ewald algorithm. Ultimately, the MD run was done with no restraint for 50 ns to evaluate the stability of structures.

### 4.5 Binding free energy

Eventually, a thorough analysis of the binding free energy (∆*G*_bind_) of all selected scaffolds was carried out using the MM‐PBSA protocol by the g‐mmpbsa package [15]. MM‐PBSA analysis gives a detailed estimation of the binding affinity of protein‐ligand interaction. To calculate the total ∆*G*_bind_, the free polar solvation energy, SASA energy, and potential energy (Van der Waals and electrostatic interactions) of each protein‐ligand complex were analyzed.

## AUTHOR CONTRIBUTIONS

**Razieh Rezaei Adriani**: Conceptualization; data curation; formal analysis; investigation; project administration; software; writing – original draft. **Seyed Latif Mousavi Gargari**: Funding acquisition; supervision; validation; writing – review & editing. **Hamid Bakherad**: Conceptualization; writing – review & editing. **Jafar Amani**: Data curation; writing – review & editing.

## CONFLICT OF INTEREST STATEMENT

The authors Razieh Rezaei Adriani, Seyed Latif Mousavi Gargari, Hamid Bakherad, and Jafar Amani declare that there are no conflicts of interest.

## ETHICS STATEMENT

This article does not contain any studies with human or animal materials performed by any of the authors.

## Supporting information

Supporting Information S1

## Data Availability

All data generated or analyzed during this study are included in this published article.
