# Supplementary material for: In silico designing and optimization of anti‐epidermal growth factor receptor scaffolds by complementary‐determining regions‐grafting technique
Source: Quant Biol. 2024 Jul 10;12(3):301–12. doi: 10.1002/qub2.63 (PMC12806330; doi:10.1002/qub2.63)
Supplement: Supplementary file 1 — Supporting Information S1 [file QUB2-12-301-s001.pdf]

>scFv/panitumumab monoclonal antibody

QVQLQESGPGLVKPSETLSLTCTVSGGSVSSGDYYWTWIRQSPGKGLEWIGHIYYSGNTNYNPSLKSRLTISIDT
SKTQFSLKLSSVTAADTAIYYCVRDRVTGAFDIWGQGTMVTVSSASTKGPSVFPLAPCSRSTSESTAALGCLVKD
YFPEPVTVSWNSGALTSGVHTFPAVLQSSGLYSLSSVVTVPSSNFGTQTYTCNVDHKPSNTKVDKTVERKGGG
GSGGGGSGGGGSDIQMTQSPSSLSASVGDRVTITCQASQDISNYLNWYQQKPGKAPKLLIYDASNLETGVPSRF
SGSGSGTDFTFTISSLQPEDIATYFCQHFDHLPLAFGGGTKVEIKRTVAAPSVFIFPPSDEQLKSGTASVVCLLNNF
YPREAKVQWKVDNALQSGNSQESVTEQDSKDSTYSLSSTLTLSKADYEKHKVYACEVTHQGLSSPVTKSFNRG
ECHHHHHH

>Scaf1

QDSTSDLIPAPPLSKVPLQQNFQDNQFHGKWYVVGLAFCQHFDHLPLAFGYATIYELKEDKSYNVTSVLFRKKKCD
YWIATFVPGSQPGEFTLVRDRVTGAFDIWGTSYLVRVVSTNYNQHAMVFFKHIYYSGNTNFAITIYGRTKELASELK
ENFIRFSKSLGLPENHIVFPVPIDQCIDG

>Scaf2

VSDVPRDLEVVAATPTSLLISWVRDRVTGAFDIWGYYRITYGETGGNSPVQEFTVFCQHFDHLPLAFGATISGLKPG
VDYTITVYAHIYYSGNTNISINYRT

>Scaf3

QVQLVESGGGSVQAGGSLRLSCTASGGSEFCQHFDHLPLAFGWFRQAPGQEREAVAVRDRVTGAFDIWRFTISR
DNAKNTVTLQMNNLKPEDTAIYYCAAHIYYSGNTNWGQGTQVTVSS

Fig.S1 The sequence of three selected scaffolds, Scaf1, Scaf2, Scaf3, and scFv

```
>5SX4_1|Chains A[auth I], C[auth L]|Panitumumab Fab Light Chain|Homo
sapiens (9606)
DIQMTQSPSSLSASVGDRVTITCQASQDISNYLNWYQQKPGKAPKLLIYDASNLETGVPSRFSGSGSG
TDFTFTISSLQPEDIATYFCQHFDHLPLAFGGGTKVEIKRTVAAPSVFIFPPSDEQLKSGTASVVCLL
NNFYPREAKVQWKVDNALQSGNSQESVTEQDSKDSTYSLSSTLTLSKADYEKHKVYACEVTHQGLSSP
VTKSFNRGEC
>5SX4_2|Chains B[auth H], D[auth J]|Panitumumab Fab Heavy Chain|Homo
sapiens (9606)
QVQLQESGPGLVKPSETLSLTCTVSGGSVSSGDYYWTWIRQSPGKGLEWIGHIYYSGNTNYNPSLKSR
LTISIDTSKTQFSLKLSSVTAADTAIYYCVRDRVTGAFDIWGQGTMVTVSSASTKGPSVFPLAPCSRS
TSESTAALGCLVKDYFPEPVTVSWNSGALTSGVHTFPAVLQSSGLYSLSSVVTVPSSNFGTQTYTCNV
DHKPSNTKVDKTVERKC
```

```
chain_type= H
{'CDR3': 'VRDRVTGAFDIWG', 'CDR2': 'HIYYSGNTN', 'CDR1': 'VSGGSVSSGDYYW'}
--- CDR1 + 2 residue per side ---
# ParaPred annotation of VSGGSVSSGDYYW
V 0.0040250253
S 0.061050057
G 0.0766101
G 0.1144763
S 0.1377744
V 0.19720979
S 0.55151266
S 0.7842272
G 0.6826688
D 0.8457368
Y 0.69999355
Y 0.9278005
W 0.017732289
----------------------------------
--- CDR2 + 2 residue per side ---
# ParaPred annotation of HIYYSGNTN
H 0.8217725
I 0.064636536
Y 0.9522733
Y 0.64375484
S 0.8850263
G 0.32782826
N 0.8982496
T 0.70342624
N 0.8133199
----------------------------------
--- CDR3 + 2 residue per side ---
# ParaPred annotation of VRDRVTGAFDIWG
V 0.037561916
R 0.5309615
D 0.836848
R 0.91570854
V 0.960553
T 0.9251448
G 0.91722876
A 0.523367
F 0.19122283
D 0.4381896
I 0.10513829
W 0.0170913
G 0.0061037005
----------------------------------


chain_type= L
{'CDR3': 'FCQHFDHLPLAFG', 'CDR2': 'IYDASNLETGV', 'CDR1': 'TCQASQDISNYLNWY'}
--- CDR1 + 2 residue per side ---
# ParaPred annotation of TCQASQDISNYLNWY
T 0.0074588074
C 0.0018127227
Q 0.011402235
A 0.0019313423
S 0.051009502
Q 0.29103553
D 0.32969385
I 0.24626851
```

```
S 0.80207276
N 0.70317924
Y 0.97880393
L 0.017167944
N 0.16149004
W 0.0037553834
Y 0.010761467
----------------------------------
--- CDR2 + 2 residue per side ---
# ParaPred annotation of IYDASNLETGV
I 0.036204495
Y 0.55487925
D 0.7031331
A 0.028350135
S 0.18454811
N 0.55149025
L 0.21064584
E 0.40853393
T 0.49088815
G 0.06696231
V 0.04140713
----------------------------------
--- CDR3 + 2 residue per side ---
# ParaPred annotation of FCQHFDHLPLAFG
F 0.010367389
C 0.0034372753
Q 0.018602395
H 0.033084605
F 0.93090385
D 0.8261948
H 0.88441646
L 0.8029969
P 0.09461637
L 0.47402692
A 0.0038748996
F 0.0050852913
G 0.0017325488
```

Fig.S2 The Sequences of heavy and light chains of panitumumab antibody and corresponding CDRs sequences resulted from Parapred software
